# Association between family poverty income ratio and Parkinson’s disease in adults aged 40 and older: a study from NHANES 2003–2020

**DOI:** 10.3389/fneur.2025.1552139

**Published:** 2025-06-05

**Authors:** Cheng Li, Jun Huang, Yemin Zhang

**Affiliations:** Department of Neurology, The Affiliated Chaohu Hospital of Anhui Medical University, Hefei, China

**Keywords:** Parkinson, NHANES, family poverty income ratio, socio-economic status, neurodegenerative disease

## Abstract

**Background:**

Parkinson’s disease (PD) is a prevalent neurodegenerative condition that has significant effects on public health. This study examines the relationship between the family poverty income ratio (PIR) and the prevalence of PD among American adults aged ≥40 years.

**Methods:**

This study leverages data from eight consecutive cycles (2003–2004 to 2017–2020) of the National Health and Nutrition Examination Survey (NHANES), a nationally representative cross-sectional surveillance program that employs stratified multistage probability sampling. The analytical cohort comprises 30,039 U.S. adults aged ≥40 years after applying exclusion criteria. To investigate the relationships between PD, PIR, and other covariates, weighted univariable logistic regression is utilized. The association between PIR and PD is then further assessed using weighted multivariable logistic regression. The possible linear or nonlinear character of this association is investigated using smoothed curve fitting. To evaluate the consistency of the association between PIR and PD across different clinical and demographic subgroups, subgroup analyses are conducted.

**Results:**

A total of the 30,039 participants in the study, 14,743 are men (49.08%) and 15,296 are women (50.92%). With an odds ratio of 0.83 (95% confidence interval: 0.75–0.91, *p* = 0.0003), PIR was found to be negatively associated with PD after controlling for all other variables. Subgroup analyses are stratified by gender, marital status, body mass index, diabetes, hypertension, stroke, and smoking status. These analyses reveal no statistically significant inverse association between the PIR and PD. However, race, age, and educational attainment exhibit significant interaction effects (*p* for interaction < 0.05), suggesting these variables may influence the PIR-PD relationship.

**Conclusion:**

Among American adults aged ≥40, this study shows a significant inverse relationship between PIR and the prevalence of PD. The results highlight how socioeconomic status may have an impact on the emergence of neurodegenerative diseases. To fully understand the intricate relationship between socioeconomic factors and PD, more extensive prospective studies are necessary.

## 1 Introduction

Parkinson’s disease (PD) is a common neurodegenerative condition that mainly affects people over 65 and whose incidence gradually rises with age (1). It is projected that more than 12 million people worldwide will have PD by 2040 (2). Significantly, PD is not just found in the elderly; a sizable portion of people under 50 are also diagnosed with the illness (2). Degeneration of dopaminergic neurons in the substantia nigra and the buildup of misfolded proteins in Lewy bodies and Lewy neurites are hallmarks of PD pathophysiology (3). Both motor and non-motor symptoms can be seen in the clinical presentation of PD. Resting tremor, bradykinesia, and muscle rigidity are examples of motor symptoms, whereas sleep disturbances, autonomic dysfunction, neuropsychological and cognitive impairments, gastrointestinal problems, visual disturbances, and exhaustion are examples of non-motor symptoms (4).

PD has become more common as the world’s population ages, placing a heavy financial strain on families and society as a whole. PD is acknowledged as a significant public health concern in lower-income populations, despite the fact that it is frequently thought to be more common in populations with higher socioeconomic status (SES) (5). SES and disease risk are strongly correlated, with those with lower SES typically having higher risk because of their lifetime exposure to harmful health determinants (6). Cardiometabolic, respiratory, psychiatric, and musculoskeletal disorders are more common in people with lower socioeconomic status (7). Household income is frequently used to measure SES and has a significant impact on health outcomes. Research has indicated that neurological development may be negatively impacted by lower socioeconomic status and limited income (8).

In addition, family income has been linked to neurological condition susceptibility: people from high-income households are less likely to suffer from ischemic stroke and Alzheimer’s disease, but there are no significant correlations between income and the risk of cerebral aneurysm, epilepsy, intracranial hematoma, or subarachnoid hemorrhage (9). PD has a complex etiology that involves interactions between lifestyle, environmental, and genetic factors. Unhealthy eating habits, insufficient nutritional intake, obesity, physical inactivity, and lifestyle choices like smoking may all contribute to the high burden of PD-related disability in less developed nations (10, 11). Higher education levels seemed to lower the risk, while diabetes, thyroid dysfunction, and gender were linked to increased risk in a survey of 24,000 Italian citizens looking into risk factors for PD. Age, coffee intake, daily physical activity, and hypertension are just a few of the numerous influencing factors that may have complex or nonlinear effects on the onset of PD (12). We used data from the National Health and Nutrition Examination Survey (NHANES) from 2003 to 2020 to examine the relationship between PD and the PIR in a sizable, representative sample of American adults aged 40 and over, considering the paucity of cross-sectional studies evaluating this relationship.

## 2 Method

### 2.1 Sources of data

The NHANES, a program created to evaluate the dietary patterns and physical health of the American people through physical examinations and interviews conducted every two years, provided the data for this study. The National Center for Health Statistics’ (NCHS) Research Ethics Review Board authorized the NHANES study, and all participants gave their informed consent in compliance with the Declaration of Helsinki’s principles.

### 2.2 Study and design

**Figure 1:**
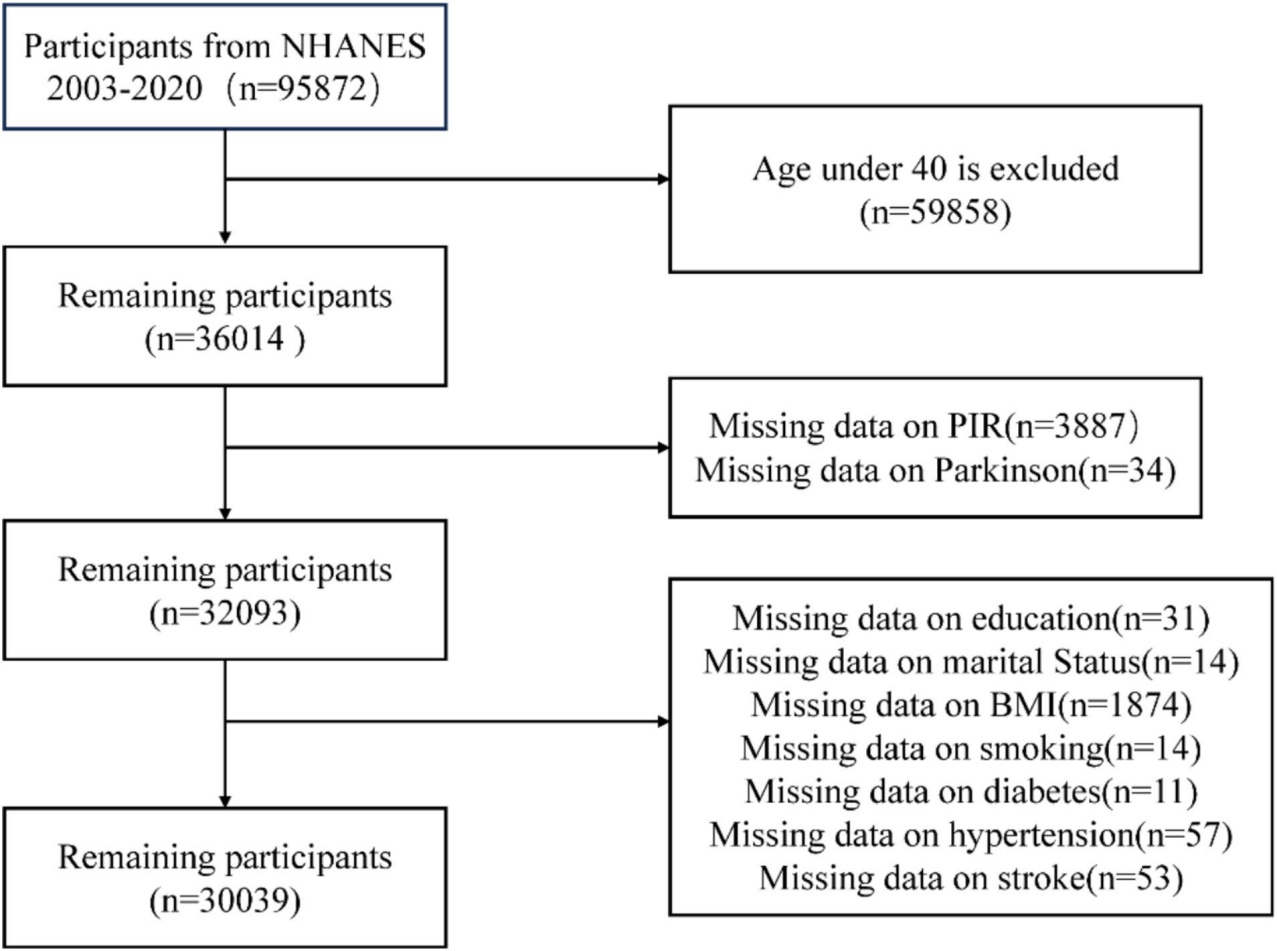
Flow diagram for selecting eligible participants from the NHANES.

NHANES data from 2003 to 2020, which included an initial sample of 95,872 participants, were used in this cross-sectional study. Participants under 40 (*n* = 59,858), those with missing PIR data (*n* = 3,887), those with missing information about the diagnosis of PD (*n* = 34), those without educational records (*n* = 31), those without marital status records (*n* = 14), those without BMI records (*n* = 1,874), those without smoking history records (*n* = 14), and those without diabetes (*n* = 11), hypertension (*n* = 57), and stroke (*n* = 54) were among the exclusion criteria. 30,039 participants were left for the final analysis after these exclusions were applied (Figure 1).

### 2.3 Study variables

The use of “anti-Parkinsonian drugs” recorded in the prescription data was used to determine whether PD was present (13, 14). Only those receiving anti-Parkinsonian medication treatment were classified as having PD; those not receiving such treatment were classified as not having the disease. This identification method is limited by the specific medications and code available in the NHANES dataset. Calculated by dividing family (or individual) income by the appropriate poverty threshold for the survey year, the family income to poverty ratio (PIR) served as a gauge of socioeconomic status. Low income (PIR < 1), middle income (PIR 1–4), and high income (PIR > 4) were the three PIR levels (15).

### 2.4 Covariates

Gender, age, marital status (divorced, cohabiting, married, unmarried, separated, or widowed), ethnicity (Mexican American, non-Hispanic Black, non-Hispanic White, other Hispanic, and other ethnicities), and educational attainment (less than 9th grade, 9th to 11th grade, high school graduate, college, or postsecondary education) were among the covariates in this study. Weight in kilograms divided by height in meters squared (kg/m^2^) was used to calculate the body mass index (BMI). Self-reported answers to the question, “Have you smoked at least 100 cigarettes in your lifetime?” were used to determine smoking status. Self-reports of having been informed by a healthcare professional that the respondent had diabetes, hypertension, or stroke were used to identify diagnoses.

### 2.5 Statistical analysis

Categorical variables were reported as frequency and percentage, and continuous variables as mean ± standard deviation. The relationship between PIR and PD was examined using a weighted multivariate logistic regression model, which included smoothed curve fitting to examine any possible nonlinear relationships. Three models were created: Model 1 was left unaltered, Model 2 was modified to account for age, gender, and race, and Model 3 was further modified to account for diabetes, hypertension, stroke, education, BMI, marital status, and smoking status. Likelihood ratio tests were used to analyze interactions. The formula for the base model was PD ~ PIR + demographic characteristics + health indicators + age + interaction term. Sex, race, marital status, and education level were among the demographic variables, and BMI, smoking, stroke, diabetes, and hypertension status were among the health indicators. To evaluate possible effect modification across age, sex, BMI, diabetes, hypertension, stroke, and smoking status categories, subgroup analyses were then conducted. R software (version 4.4.1) and EmpowerStats (version 4.0) were used for all statistical analyses, and a *p* value < 0.05 was deemed statistically significant.

## 3 Results

### 3.1 Demographic characteristics of participants

A total of the 30,039 participants in the statistical analysis, 389 had a diagnosis of PD, while 29,650 were categorized as participants without PD. PD patients were more likely to be older, female, have a higher body mass index, be non-Hispanic White, and have a lower family income than non-PD patients (Table 1).

**Table 1:** Basic characteristics of participants.

Characteristic	Total	Non-PD	PD	*p*-value
Age	57.63 ± 0.15	57.58 ± 0.14	61.55 ± 0.91	< 0.0001*
PIR	3.20 ± 0.03	3.20 ± 0.03	2.68 ± 0.12	< 0.0001*
BMI (kg/m^2^)	29.41 ± 0.07	29.40 ± 0.07	30.47 ± 0.44	0.02*
Gender				0.05
Female	52.64	52.55	59.56	
Male	47.36	47.45	40.44	
Ethnicity				< 0.001*
Mexican American	5.87	5.89	3.88	
Other Hispanic	4.32	4.33	3.08	
Non-Hispanic White	72.69	72.57	82.18	
Non-Hispanic Black	10.24	10.26	8.07	
Other Race	6.89	6.94	2.79	
Married				0.03*
Never married	6.82	6.78	10.36	
Married/living with partner	67.74	67.83	60.58	
Widowed/divorced/separated	25.44	25.40	29.06	
Education				0.03*
Less than 9th grade	6.05	6.04	6.45	
9-11th grade	9.95	9.92	12.42	
High school graduate or GED	24.54	24.52	25.86	
Some college or AA degree	29.84	29.78	35.16	
College graduate or above	29.62	29.74	20.12	
Smoking status				0.62
No	51.60	51.58	53.05	
Yes	48.40	48.42	46.95	
Stroke				< 0.0001*
No	95.66	95.76	87.67	
Yes	4.34	4.24	12.33	
Diabetes				0.01*
No	83.47	83.57	75.56	
Yes	13.78	13.69	20.83	
Borderline	2.75	2.74	3.61	
Hypertension				< 0.0001*
No	57.08	57.27	41.98	
Yes	42.92	42.73	58.02	

Mean±SD for continuous variables, and *P* value calculated by weighted *t* test.

% for categorical variables, and *P* value calculated by weighted Chi-square test.

PIR, poverty income ratio; PD, Parkinson’s disease; BMI, body mass index; *p < 0.05 was considered statistically significant.

### 3.2 Univariate regression analysis between PIR and PD

Significant correlations were found between PD and a number of variables in the entire study population, including age, non-Hispanic White ethnicity, marital status (married or cohabiting), higher educational attainment (college and above), BMI, stroke, diabetes mellitus, hypertension, and income level (*p* < 0.05). The prevalence of PD was positively correlated with age [OR = 1.67, 95% CI: (1.29, 2.16)]. The odds of PD were significantly higher for patients with a history of stroke or diabetes than for those without these conditions [OR = 3.18, 95% CI: (2.13, 4.74) and OR = 1.68, 95% CI: (1.21, 2.33), respectively]. Likewise, PD risk was higher in hypertensive individuals than in non-hypertensive individuals [OR = 1.85, 95% CI: (1.42, 2.42)]. On the other hand, those with greater incomes had a noticeably lower risk of PD [OR = 0.42, 95% CI: (0.28, 0.63)]. (Table 2).

**Table 2:** Analysis of univariate logistics regressions of PIR and PD.

Variables	OR (95%CI)	P-value
Age (years)		
<=65	Ref.	
>65	1.67 (1.29, 2.16)	0.0001*
Gender		
Female	Ref.	
Male	0.75 (0.57, 1.00)	0.0511
Ethnicity		
Mexican American	Ref.	
Other Hispanic	1.08 (0.60, 1.95)	0.7963
Non-Hispanic White	1.72 (1.20, 2.46)	0.0034*
Non-Hispanic Black	1.20 (0.77, 1.86)	0.4318
Other Race	0.61 (0.28, 1.35)	0.2252
Married		
Never married	Ref.	
Married/living with partner	0.58 (0.38, 0.90)	0.0153*
Widowed/divorced/separated	0.75 (0.48, 1.16)	0.1931
Education		
Less than 9th grade	Ref.	
9-11th grade	1.17 (0.75, 1.84)	0.4892
High school graduate or GED	0.99 (0.62, 1.57)	0.9600
Some college or AA degree	1.11 (0.77, 1.59)	0.5865
College graduate or above	0.63 (0.41, 0.97)	0.0374*
BMI (kg/m^2^)	1.02 (1.01, 1.04)	0.0086*
Smoking status		
No	Ref.	
Yes	0.94 (0.75, 1.19)	0.6178
Stroke		
No	Ref.	
Yes	3.18 (2.13, 4.74)	<0.0001*
Diabetes		
No	Ref.	
Yes	1.68 (1.21, 2.33)	0.0022*
Borderline	1.46 (0.64, 3.29)	0.3670
Hypertension		
No	Ref.	
Yes	1.85 (1.42, 2.42)	<0.0001*
PIR		
<=1	Ref.	
1–4	0.81 (0.57, 1.16)	0.2571
>4	0.42 (0.28, 0.63)	0.0001*

OR: odds ratio; CI: confidence interval. *p < 0.05 was considered statistically significant.

### 3.3 Multivariate regression analysis of the relationship between PIR and PD

All three models weighted multivariate logistic regression analysis showed a consistent negative correlation between PIR and PD. The odds ratio was 0.82 [95% CI: (0.76–0.94)] in Model 1 (unadjusted); 0.80 [95% CI: (0.75–0.96)] in Model 2 (adjusted for age, gender, and race); and 0.83 [95% CI: (0.75–0.91)] in Model 3 (fully adjusted for age, gender, race, education, BMI, marital status, smoking, diabetes, hypertension, and stroke). The high-income group had a significantly lower risk of PD according to additional analysis that treated PIR as a categorical variable (PIR > 4). Participants in Q2 (PIR 1.2–2.24), Q3 (PIR 2.24–4.28), and Q4 (PIR > 4.28) all displayed a statistically significant inverse association with PD when PIR was split into quartiles, in contrast to those in Q1 (PIR < 1.2) (Table 3).

**Table 3:** The associations between family PIR and PD.

Exposure	Model1OR (95%CI) *P*-value	Model2OR (95%CI) *P*-value	Model3OR (95%CI) *P*-value
PIR	0.82 (0.76, 0.94) < 0.0001*	0.80 (0.75, 0.96) < 0.0001*	0.83 (0.75, 0.91) 0.0003*
PIR classification			
Low income (PIR ≤ 1)	Reference	Reference	Reference
Middle income (PIR 1–4)	0.81 (0.57, 1.16) 0.2570	0.67 (0.47, 0.96) 0.0286*	0.71 (0.50, 1.00) 0.0568
Affluent (PIR > 4)	0.41 (0.27, 0.63) < 0.0001*	0.35 (0.23, 0.55) < 0.0001*	0.41 (0.27, 0.64) 0.0001*
PIR classification			
Q1 (PIR < 1.2)	Reference	Reference	Reference
Q2 (PIR 1.2–2.24)	0.96 (0.68, 1.53) 0.8155	0.81 (0.58, 1.15) 0.2425	0.84 (0.60, 1.19) 0.34
Q3 (PIR 2.24–4.28)	0.66 (0.43, 1.02) 0.0629	0.58 (0.38, 0.88) 0.0113*	0.62 (0.41, 0.95) 0.0283*
Q4 (PIR > 4.28)	0.45 (0.30, 0.67) 0.0001*	0.39 (0.26, 0.60) < 0.0001*	0.46 (0.29, 0.71) 0.0007*
P for trend	<0.0001*	<0.0001*	<0.0001*

Model I: None covariates were adjusted. Model II: Age, gender, and race were adjusted. Model III: Age, gender, race, education, BMI, marital status, smoking status, diabetes status, hypertension status, and stroke status were adjusted. *p < 0.05 was considered statistically significant.

### 3.4 Subgroup analysis

**Figure 2:**
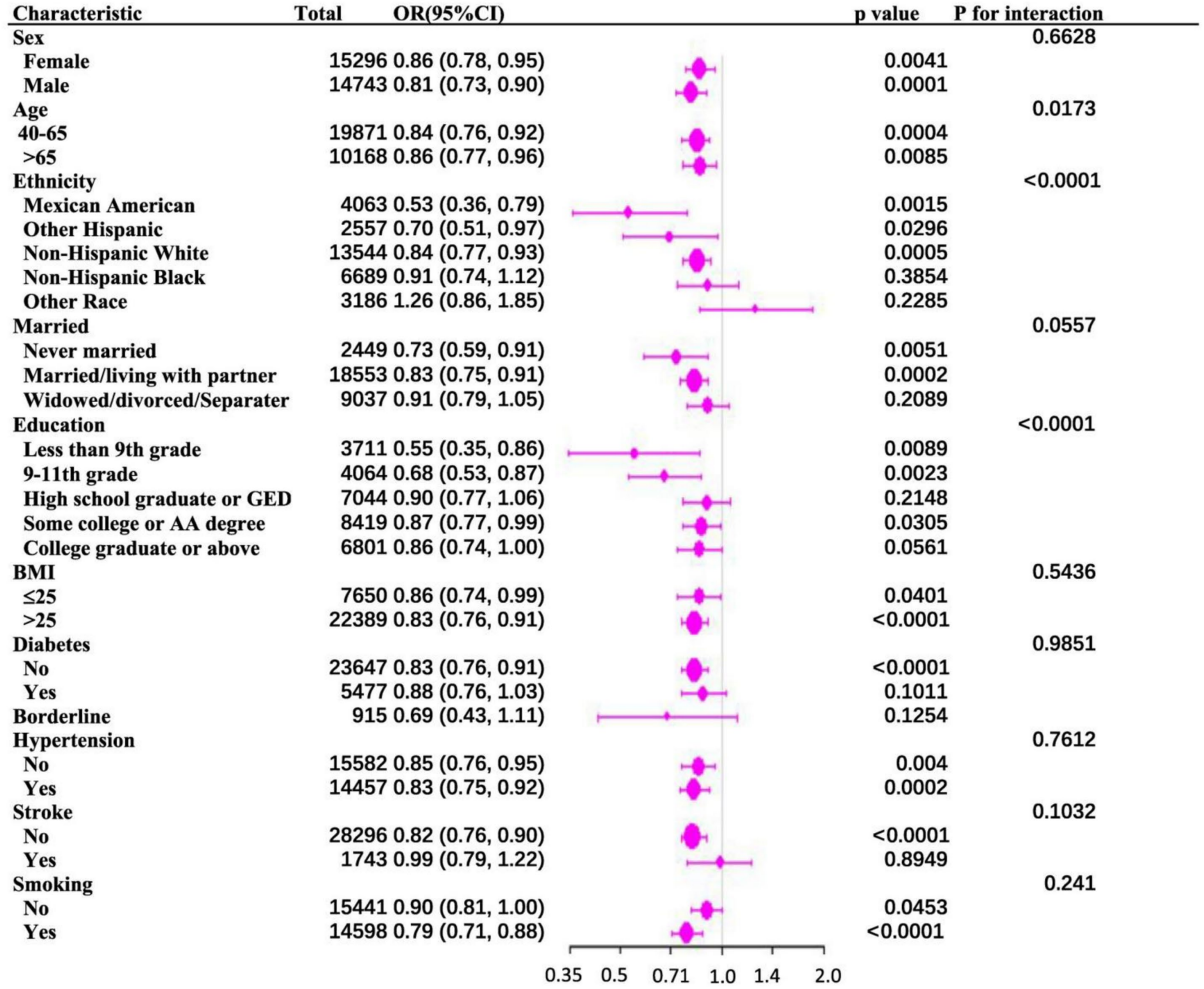
**(A)** Subgroup analysis of the association between family PIR and Parkinson’s disease. **(B)** The above model adjusted for age, gender, race, education, BMI, marital status, smoking status, diabetes status, hypertension status, and stroke status.

**Figure 3:**
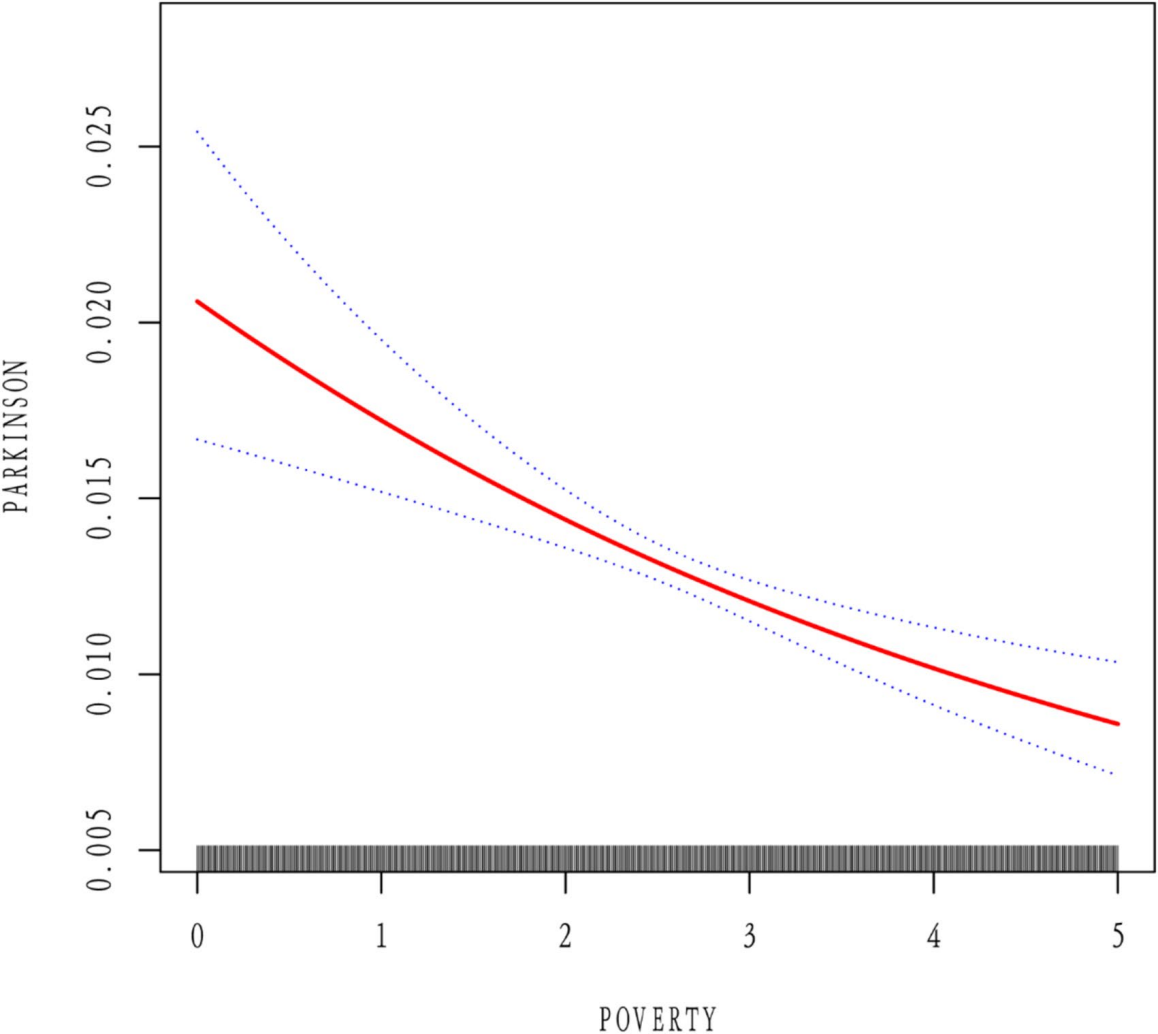
**(A)** Smooth curve fittings of the association between PIR and Parkinson’ disease. **(B)** The associations were adjusted for age, gender, race, education, BMI, marital status, smoking status, diabetes status, hypertension status, and stroke status.

**Figure 4:**
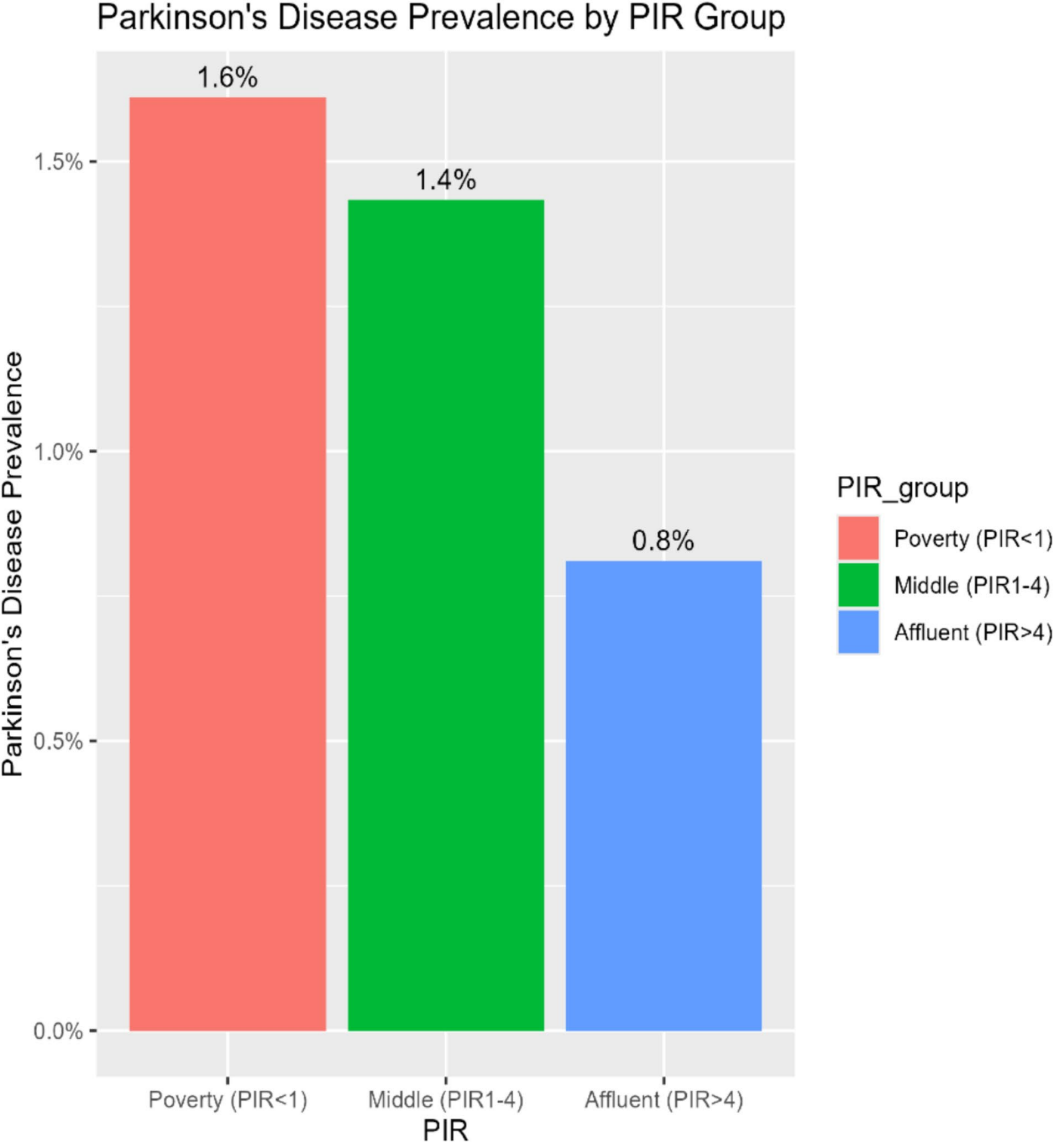
Parkinson’s disease prevalence by PIR group.

To investigate the consistency of the relationship between PIR and PD across various demographic and clinical contexts, subgroup stratification was carried out based on gender, age, race, education level, marital status, BMI, diabetes, hypertension, stroke, and smoking status. Within the subgroups based on gender, marital status, BMI, diabetes, hypertension, stroke, and smoking, the inverse relationship between PIR and PD was not statistically significant. However, there were notable interactions with age, education level, and race (*p* for interaction < 0.05). Among age-stratified groups, middle-aged adults (40–65 years) and older adults (>65 years) had a 16 and 14% lower risk of PD, respectively, for every unit increase in PIR. The middle-aged subgroup showed a stronger negative correlation between PIR and PD (*p* = 0.017) (Figure 2). A nonlinear inverse relationship between PIR and PD was also discovered through the application of a smoothed curve-fitting analysis (Figure 3). Finally, Figure 4 shows the prevalence of PD by socioeconomic group according to PIR: the highest prevalence (1.6%) was found in the lowest income group (PIR < 1), followed by the middle-income group (PIR 1–4) at 1.5%, and the lowest prevalence (0.5%) was found in the highest income group (PIR > 4).

## 4 Discussion

This study aimed to investigate the correlation between the PIR and the incidence of PD in individuals aged 40 and above in the United States. This cross-sectional analysis of 30,039 participants revealed that individuals with elevated PIR values were significantly less likely to develop PD. A strong inverse correlation between PIR and PD was observed, remaining stable across fully adjusted models. Subgroup analyses indicated significant interactions between PIR and PD concerning race, age, and educational attainment; however, no significant interaction effects were found for gender, marital status, BMI, diabetes, hypertension, stroke, or smoking status.

PD is affected by various environmental and biological factors. Epidemiological studies have shown that exposure to insecticides, low-frequency magnetic fields, solvents, heavy metals, chemical agents, and air pollutants represents significant risk factors for PD, especially in developing and middle-income nations. These exposures may elicit pathological alterations in alpha-synuclein proteins within the olfactory and gastrointestinal systems, which subsequently disseminate through parasympathetic and sympathetic neural pathways, ultimately resulting in the onset of Lewy body disorders and PD (16, 17). In addition, the advancement of PD can be intensified by risk factors including inadequate access to medical resources, inequities in healthcare provision, detrimental lifestyle choices, and psychosocial stressors (18). Socioeconomic disparities may induce chronic psychological stress, which can impair 5-hydroxytryptaminergic transmission and noradrenergic activity in brain areas such as the prefrontal cortex, striatum, and hippocampus, thus influencing the pathophysiology of PD (19).

Socioeconomic disparities can profoundly influence disease risk among populations. Individuals from affluent households may experience advantages from prompt identification and management of disease symptoms, whereas those from economically disadvantaged groups may encounter increased risk due to restricted awareness, healthcare accessibility, and preventive resources (20). However, previous research regarding the correlation between household income and the prevalence of PD has yielded inconsistent results (10). One study indicated a reduced prevalence of PD among individuals with lower household incomes, in contrast to another investigation that found a significantly lower PIR in individuals with PD compared to those without the condition (2.68 ± 0.12), implying a negative correlation between PIR and PD. Multivariable regression analyses, controlled for potential confounders, further corroborated this inverse relationship. Lisa et al. documented analogous results in a Canadian cohort, indicating that lower SES correlated with elevated prevalence and incidence rates of PD. Urban low-income populations demonstrated a greater disease burden than their high-income counterparts (21). A Korean national cohort study similarly revealed that diminished individual-level socioeconomic status, characterized by income and health insurance type, was inversely correlated with PD mortality, while regional socioeconomic indicators, such as residential area, showed no significant association with PD mortality (22).

The stratified analysis of this study revealed the association between educational attainment and PD risk. The data demonstrated a statistically significant reduction in PD prevalence among individuals with university-level or higher education compared to those with lower educational levels (*p* < 0.05). Existing literature suggests that higher education may influence disease progression through dual neuroprotective mechanisms: first, cognitive reserve acquired through education may delay clinical manifestation by enhancing neural compensatory functions (23); second, neuroimaging evidence indicates that prolonged educational experience can induce structural neuroplasticity, while epigenomic studies suggest its potential regulatory effect on CGG repeat expansion in fragile X messenger ribonucleoprotein 1 (FMR1) gene, thereby enhancing neuronal resistance to degenerative processes (24, 25). Moreover, education may exert indirect neuroprotective effects by modulating lifestyle and socioeconomic status – individuals with higher educational attainment exhibit less severe motor dysfunction. This enhanced clinical resilience to PD pathology-induced motor impairments likely originates from the synergistic effects of multiple education-associated mechanisms, including strengthened synaptic plasticity, optimized brain network efficiency, and activation of neural regenerative mechanisms (26). However, the precise dose–response relationship between educational attainment and PD risk, along with its mechanistic pathways, requires validation through multicenter prospective cohort studies. In age-stratified analysis, the incidence rate showed a progressive increase among patients aged over 65 years (27), potentially associated with age-related blood–brain barrier dysfunction and cumulative mitochondrial impairment (28). No statistically significant interactions were observed in subgroups stratified by sex, BMI, marital status, diabetes, hypertension, stroke, or smoking status, possibly due to insufficient statistical power to detect moderate effect sizes in current analysis, as well as subgroup heterogeneity and confounding factors. Longitudinal studies have indicated that smoking, alcohol consumption, and high cholesterol exhibit protective effects against PD, while no association was observed between BMI and PD risk (29). The relationship between diabetes and PD remains controversial. Diabetes and PD may share common cellular mechanisms, including mitochondrial dysfunction and underexpression of the transcriptional regulator PPARγ coactivator 1α (PGC1α), which impairs mitochondrial biogenesis and respiratory function (29).

This research possesses multiple limitations. First, as a cross-sectional study, the study precludes causal inference regarding the observed association between PIR and PD. The temporality between socioeconomic exposure and PD onset cannot be ascertained, and potential mechanistic pathways remain speculative. Additionally, participants were diagnosed with PD solely based on whether they were taking Parkinson’s medication. This approach lacks diagnostic specificity, as antiparkinsonian drugs are prescribed for other movement disorders, potentially inflating false-positive rates. Conversely, untreated early-stage PD cases or those with atypical presentations may be systematically excluded, leading to outcome misclassification and attenuated effect estimates. Finally, as an independent socioeconomic indicator, the PIR has limitations in accurately reflecting an individual’s specific likelihood of disease prevalence. PIR does not account for regional cost-of-living variations or temporal income fluctuations, limiting its granularity in characterizing individual-level socioeconomic vulnerability. Our findings reveal a notable nonlinear inverse relationship between PIR and PD, indicating that PIR may serve as a predictive tool for identifying populations at elevated risk for the condition.

## 5 Conclusion

PIR is strongly correlated with the occurrence of PD in individuals aged over 40 in the United States. A diminished PIR is associated with a heightened probability of developing PD, suggesting that PIR may function as a significant predictive indicator for the risk of disease onset. These findings underscore the potential benefits of enacting proactive measures to alleviate disease progression, including elevating income levels in economically disadvantaged areas and augmenting early screening and detection initiatives for PD.

## Data Availability

The datasets presented in this study can be found in online repositories. The names of the repository/repositories and accession number(s) can be found in the article/supplementary material.
